# A Mature NK Profile at the Time of HIV Primary Infection Is Associated with an Early Response to cART

**DOI:** 10.3389/fimmu.2017.00054

**Published:** 2017-02-10

**Authors:** Françoise Gondois-Rey, Antoine Chéret, Françoise Mallet, Ghislain Bidaut, Samuel Granjeaud, Camille Lécuroux, Mickaël Ploquin, Michaela Müller-Trutwin, Christine Rouzioux, Véronique Avettand-Fenoël, Andrea De Maria, Gilles Pialoux, Cécile Goujard, Laurence Meyer, Daniel Olive

**Affiliations:** ^1^CNRS, INSERM, Institut Paoli-Calmettes, CRCM, Immunity and Cancer Team, Aix Marseille Univ, Marseille, France; ^2^APHP, Hôpital Bicêtre, Internal Medecin Unit, Le Kremlin-Bicêtre, France; ^3^U1184, Paris-Sud Univ, Le Kremlin-Bicêtre, France; ^4^CEA, DSV/iMETI, IDMIT, Fontenay-aux-Roses, France; ^5^INSERM U1184, ImVA “Immunology of Chronic Viral Infections and Autoimmune Diseases”, Le Kremlin-Bicêtre, France; ^6^Institut Pasteur, HIV, Inflammation and Persistance Unit, Paris, France; ^7^Virology Laboratory, APHP CHU Necker-Enfants Malades, Paris, France; ^8^CNRS, INSERM, Institut Paoli-Calmettes, CRCM, CiBi Platform, Aix Marseille Univ, Marseille, France; ^9^EA 7327 Paris Descartes University, Paris, France; ^10^Dipartimento di Scienze della Salute and Centro di Eccellenza per la Ricerca Biomedica, Università di Genova, Genova, Italy; ^11^Clinica Malattie Infettive, IRCCS Azienda Ospedaliera Universitaria San Martino – Istituto Nazionale per la Ricerca sul Cancro, Genova, Italy; ^12^APHP, Hôpital Tenon, Infectious Diseases Unit, Paris, France; ^13^INSERM, CESP U1018, Epidemiology and population health, APHP, Hôpital Bicêtre, Paris 11 Univ, Le Kremlin-Bicêtre, France

**Keywords:** HIV, primary infection, NK cells maturation, cART, memory-like NK

## Abstract

Natural killer (NK) cells are major effectors of the innate immune response. Despite an overall defect in their function associated with chronic human immunodeficiency virus (HIV) infection, their role in primary HIV infection is poorly understood. We investigated the modifications of the NK cell compartment in patients from the ANRS-147-Optiprim trial, a study designed to examine the benefits of intensive combination antiretroviral therapy (cART) in patients with acute or early primary HIV infection. Multiparametric flow cytometry combined with bioinformatics analyses identified the NK phenotypes in blood samples from 30 primary HIV-infected patients collected at inclusion and after 3 months of cART. NK phenotypes were revealed by co-expression of CD56/CD16/NKG2A/NKG2C and CD57, five markers known to delineate stages of NK maturation. Three groups of patients were formed according to their distributions of the 12 NK cell phenotypes identified. Their virological and immunological characteristics were compared along with the early outcome of cART. At inclusion, HIV-infected individuals could be grouped into those with predominantly immature/early differentiated NK cells and those with predominantly mature NK cells. Several virological and immunological markers were improved in patients with mature NK profiles, including lower HIV viral loads, lower immune activation markers on NK and dendritic cell (DC), lower levels of plasma IL-6 and IP-10, and a trend to normal DC counts. Whereas all patients showed a decrease of viremia higher than 3 log_10_ copies/ml after 3 months of treatment, patients with a mature NK profile at inclusion reached this threshold more rapidly than patients with an immature NK profile (70 vs. 38%). In conclusion, a better early response to cART is observed in patients whose NK profile is skewed to maturation at inclusion. Whether the mature NK cells contributed directly or indirectly to HIV control through a better immune environment under cART is unknown. The NK maturation status of primary infected patients should be considered as a relevant marker of an immune process contributing to the early outcome of cART that could help in the management of HIV-infected patients.

## Introduction

Natural killer (NK) cells are one of the major innate immune components involved in the rapid response of the host to invading virus (1). Their function is probably crucial at the time of infection and can impact the quality of adaptive immune responses and the overall outcome of infections. NK cell activity is regulated by activating and inhibitory receptors, but their effector functions are intrinsically linked to their maturation (2). Cytolysis is the typical NK cell function, but NK cell also play an antiviral role through the release of soluble factors, such as IFN-γ and TNF-α (3), which activate T cells, macrophages, and dendritic cells (DCs). CD56^bright^ NK cells are described as the progenitors of CD56^dim^, the latter being endowed with the main NK cell effector functions (2). CD56^dim^ cells sequentially progress from an immature population, characterized by a high degranulation and proliferation potential, to a terminally differentiated population, characterized by potent cytokine production at the expense of cell division and degranulation (4). Immature NK cells express NKG2A, a C-type lectin receptor forming an inhibitory heterodimer with CD94 to interact with HLA-E on target cells (5). HLA-E is a non-classical major histocompatibility complex (MHC) class I molecule whose expression is enhanced on infected cells through the presentation of viral peptides (6). During maturation, NKG2A loss is compensated by the acquisition of self-inhibitory killer-cell immunoglobulin-like receptor (KIR) expression, while CD57, a marker of senescence, is acquired (4). NKG2C/CD94 is the activating alternative receptor of HLA-E on NK cells (7). This receptor was initially described on a subset of NK cells expanded during CMV infection (8, 9), but recently, other viruses, including human immunodeficiency virus (HIV), were also shown to drive NKG2C^+^ NK cell expansion, in the context of CMV co-infection (10, 11). The persistence of a NKG2C^+^CD57^+^ NK cell subset for more than 1 year after CMV or Hantavirus infection has led to the proposition that they are a memory-like form of NK cell (8, 12, 13). Therefore, the co-expression of CD56, CD16, NKG2A, NKG2C, and CD57 delineates sequential stages of the NK cell maturation process suggesting the acquisition of typical effector functions.

Natural killer cell functions are affected early after HIV infection (14). In addition, many modifications of the NK cell compartment, including decrease of CD56^bright^, expansion of CD56^dim^, and appearance of a functionally compromised subset of CD56^dim^ expressing low levels of CD56 or CD16 were reported (15). Inversion of the ratio of NKG2A to NKG2C was described in primary HIV-infected patients (11, 16). In chronically infected patients, an overall increase of mature CD57^+^ NK cells was observed (17). In cohort of individualist risk of HIV infection, NK cell activation at the time of primary infection has been both positively and negatively correlated with the risk of HIV acquisition (18, 19). Recently, a correlation was demonstrated between NK cell repertoire diversity, linked to progression to maturity, and increased susceptibility to HIV infection (20). Therefore, while NK maturation seems to be an interesting parameter in HIV infection, so far, the impact of the overall maturation of NK cells on the outcome of primary HIV-infection (PHI) remains elusive.

The Optiprim trial was designed to evaluate to what extent intensive antiviral therapy started during primary HIV infection contributes to a decrease in the size of HIV reservoirs and helps to achieve a so-called posttreatment controller (PTC) status (21). A sub-study was designed to investigate innate immune parameters. Considering the important role of NK cell maturation for their effector properties, we investigated the NK cell compartment with a combination of markers known to characterize sequential steps of the NK cell differentiation pathway. Thirty primary HIV-infected patients peripheral blood mononuclear cell (PBMC) samples were investigated at inclusion and 3 months after the onset of combination antiretroviral therapy (cART). Because cell populations expressing unexpected combinations of markers might be expanded in pathological conditions, new bioinformatics methods were applied to analyze multiparametric cytometry data in an unsupervised approach (22). This allowed the identification of a relationship between profiles of NK cells skewed to immaturity or maturity and virological and immune parameters reached naturally a few weeks after infection and after early cART.

## Subjects and Methods

### Ethical Statement

All study participants provided written informed consent.

The study was approved by the Sud-Mediterranee-1 Ethics Committee and the French Health Products Safety Agency and complied with the Helsinki Declaration.

### Study Population

Human immunodeficiency virus-1-infected subjects with PHI were included in a multicenter phase 3 randomized trial (ANRS-147 OPTIPRIM) (www.ClinicalTrials.gov, number NCT01033760). The endpoint was the impact of intensive vs. standard cART at month 24 on blood HIV-DNA levels. The results of this study have been published (21). Antoine Chéret was the Principal Investigator, Laurence Meyer the Methodological Investigator, and Christine Rouzioux the Virologist Investigator. We proposed a sub-study where the participants would give blood samples at day 0 (before cART initiation) and month 3. This sub-study, in which Daniel Olive and Françoise Gondois-Rey were the investigators, was designed to investigate parameters of innate immunity linked to cART efficacy. Among 90 patients in the main study, 30 patients were randomly included in this sub-study. This work shows original data on NK cells, DC, CMV, and plasma cytokines, and uses information from the main study. Patient characteristics are listed in Table 1.

**Table 1:** **Patients’ characteristics at inclusion**.

Number of patients	30
Number of patients acutely infected	14
Time between estimated date of infection and enrollment (days)	34 (20–55)
Age (years)	39.4 (23–55)
Number of patients handled by intensive combination antiretroviral therapy	15
CD4 counts at T0 (count/μL)	550 (323–1,012)
CD8 counts at T0 (count/μL)	1,704 (417–8,157)
CD4 to CD8 ratio at T0	0.47 (0.08–1.32)
Human immunodeficiency virus (HIV)-RNA at T0 (log_10_ copies/mL)	5.51 (3.2–7)
HIV-DNA at T0 [log_10_ copies/10^6^ peripheral blood mononuclear cell (PBMC)]	3.747 (2.78–4.68)
CD4 counts at M3 (count/μL)	656 (275–1,244)
CD8 counts at M3 (count/μL)	705 (371–1,282)
HIV-RNA at M3 (log_10_ copies/mL)	1.88 (1.3–3.42)
HIV-DNA at M3 (log_10_ copies/10^6^ PBMC)	2.93 (2.25–3.62)

*Mean values and (range) are indicated*.

Fifteen healthy donor samples were obtained from the French Blood Bank (EFS, Etablissement Français du Sang) as controls. PBMC samples were frozen and kept in liquid nitrogen until tested.

### Flow Cytometry

Peripheral blood mononuclear cells were stained with multiparametric panels containing 9 or 12 fluorescent markers, respectively, designed to investigate NK and DC populations. The NK panel contained NKG2A-PacBlue (clone Z199, home-made), live–dead Aqua (Life Technology), CD57-FITC (Beckman Coulter; 1/30), NKG2C-PE (R&D; 1/40), CD14-PC5 (Beckman Coulter; 1/30), CD19-PC5 (Beckman Coulter; 1/30), CD56-PC7 (Beckman Coulter; 1/30), CD3-AF700 (BD Biosciences; 1/40), and CD16-APCH7 (BD Biosciences; 1/40). The DC panel contained live–dead Aqua (Life Technology), BDCA2-FITC (Miltényi; 1/30), CD123-PercpCy5.5 (BD Biosciences; 1/20), HLA-DR-ECD (Beckmann Coulter; 1/40), CD3-PC5 (BD Biosciences; 1/40), CD56-PC5 (Beckman Coulter; 1/30), CD19-PC5 (Beckman Coulter; 1/40), CD33-PC7 (BD Biosciences; 1/40), CD14-APCH7 (BD biosciences; 1/40), and CD16-AF700 (BD Biosciences; 1/40). Cells were incubated for 20 min at RT with reagents pre-mixed in PBS, washed, and then fixed with 4% PFA. Data were acquired on a LSRII-SORP (BD Biosciences) equipped with four lasers (405 nm/100 mW, 488 nm/100 mW, 560 nm/50 mW, and 630 nm/40 mW). Photo-multiplicators were set using unstained and fully stained samples and linked to the cytometer standardization using the acquisition setting tool. Also, 7.74 × 10^6^ ± 3 × 10^5^ events were recorded. Compensations were performed with beads individually stained with corresponding reagents.

### Flow Cytometry Data Analysis

Data were exported and analyzed with FlowJo (version 9-2, MacOS X). NK cells were gated as CD3^−^CD14^−^CD19^−^CD56^+^CD16^+^, CD3^−^CD14^−^CD19^−^CD56^+^CD16^−^, or CD3^−^CD14^−^CD19^−^CD56^−^CD16^+^ (Figure S1 in Supplementary Material). Contaminating non-classical monocytes represented less than 0.11% of the gated events (not shown). The remaining data (3.5 × 10^5^ ± 2.5 × 10^4^ events) were exported to create new files further subjected to automated gating. DC’s were defined as CD3^−^CD14^−^CD19^−^CD56^−^CD16^−^HLA-DR^+^live cells. In the DC gate, the pDC were gated as CD33^low^BDCA2^+^CD123^+^ cells, the mDC as CD33^high^BDCA2^−^CD123^−^ cells.

### Automatic Clustering

The flowClust implementation version 3.4.11on R version 3.1.2 under Linux Cent OS 6 was applied on the following parameters: CD56-PC7, CD16-APCH7, NKG2A-PacBlue, NKG2C-PE, CD57-FITC, to compute clusters as described (22). The number of clusters that fit the data optimally was estimated by computing flowClust on a predefined range and comparing them with the statistical criteria BIC and ICL (not shown). This number was estimated to be above 20; therefore, 27 clusters were computed in the events within the NK gate of the 60 samples (30 patients, T0, and M3) and one healthy donor. Two samples failed to be computed.

MFI values and event counts of the 1,593 clusters generated after computation were exported in an Excel table.

### Multiparametric Data Management with MeV

MeV (version 4.9.0, https://sourceforge.net/projects/mev-tm4/) (23) was used to visualize and group multiparametric clusters using hierarchical clustering (HCL) of their centers of CD56, CD16, CD57, NKG2A, and NKG2C MFI. Euclidean distance and average linkage were chosen. Prior to MeV, centers were rescaled to adjust the 5 (respectively, 95) percentile of each dimension to −3 (respectively, +3) and normalized. The tree was cut interactively using objective MeV tools and color interpretation of the heatmap to define populations as homogeneous groups of clusters. Names were interactively given according to the comparisons of signatures between groups and the expression of known NK cell markers. The blocks were saved and imported in a spreadsheet program, leading to a matrix with a population identifier column associated to the initial count of events. The percentages of each population of each patient sample were summarized using pivot tables.

### Virus Quantification

Human immunodeficiency virus-RNA was quantified in plasma by real-time RT-PCR with the Cobas TaqMan HIV1 v2.0 assay (Roche Diagnostics). Threshold values were arbitrarily given to samples below the threshold of the assay (20 RNA copies/mL). Total HIV-DNA was quantified by ultra-sensitive real-time PCR in PBMC using the Generic HIV-DNA assay from BioCentric (Bandol, France) as described (24).

### Cytokine Quantification

#### Plasma Cytokines

IP-10 concentrations were determined in stored plasma or serum samples (−80°C) by specific enzyme-linked immunosorbent assay, human Quantikine CXCL10 (R&D Systems, Minneapolis, MN, USA) according to the manufacturers’ instructions. Levels of IL-6 were measured in frozen plasma samples with specific ELISA assays (Human IL-6 Platinum ELISA, eBioscience). Samples with undetectable levels of IL-6 were arbitrarily attributed half the minimal detectable value (0.46 pg/mL).

### Statistics

Statistical graphics were performed with Prism 6 software. The Kruskall–Wallis test followed by multiple comparison Dunn’s posttest were used to compare variables between groups. Correlations were evaluated by using simple linear regression analysis and Spearman’s rank correlation test.

## Results

### Automatic Clustering Revealed NK Cell Differentiation Subtypes within HIV-Primary Infected Patients’ PBMCs

**Figure 1:**
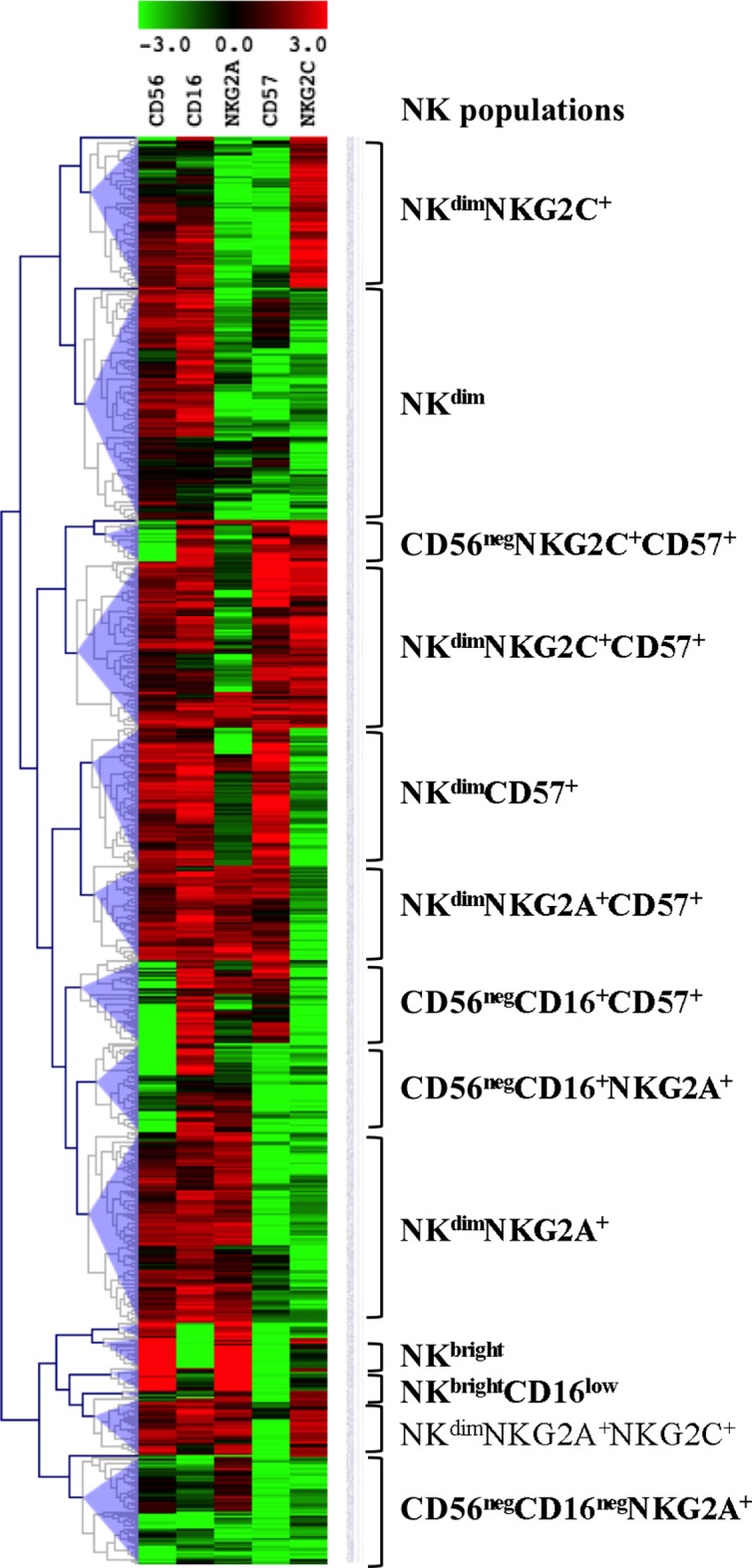
**Identification of natural killer (NK) cell populations**. Visualization of the 756 clusters computed into the NK cells files from the 28 T0 patients’ samples in a MeV heatmap. The clusters are visualized and merged according to their normalized MFI for the five markers shown on top using the MeV software hierarchical clustering tools. NK populations are defined as groups of merged clusters and annotated according to their signatures shown on the right.

We investigated the phenotypes of differentiation of NK cells with multiparametric cytometry using CD56, CD16, NKG2A, CD57, and NKG2C, known to delineate sequential stages of NK cell maturation (2, 4). In order to discover unexpected populations, multi-stained samples were analyzed through unsupervised computation of clusters, using a method previously validated (22). Computed clusters were visualized in a MeV heatmap according to their normalized MFI for the five markers and merged to identify populations as homogeneous groups of clusters (Figure 1). The tree was interactively cut to summarize the results in 19 groups, using objective tools of MeV and a subjective overview of the heatmap (Figure 1). Six of them including only a few clusters were excluded because of their rare representation. Thirteen groups were named according to comparisons of marker MFI and homogeneity with known NK population signatures, a process similar to manual gating of cytometry data visualized in dot plots (Figure 1). Accordingly, two groups of clusters expressing the highest levels of CD56, of NKG2A, no or low levels of CD16, a typical CD56^bright^ signature, were thus named. Seven populations expressing medium levels of CD56 and high levels of CD16 were identified as CD56^dim^. They included several groups differentially expressing NKG2A and CD57, two markers linked to immaturity or maturity, respectively, and NKG2C, a NK cell receptor expanded during CMV infections. Four populations expressing low levels of CD56 or CD16 previously described in HIV-infected patients as dysfunctional NK populations were identified (15). Finally, phenotypes of NK cells usually found in human PBMC, including populations specifically expanded during HIV infection, and new phenotypes characterized by unexpected combinations of markers were found in patients’ samples using the automatic clustering and interactive merging approach. It should be emphasized that other analyses of the same data, based on the choice of higher or lower numbers of populations could have also been pertinent. As an example, the few clusters characterized by CD56^bright^CD16^neg^NKG2C^+^ visible at the bottom of the tree were included in the CD56^bright^ pool while other cuts of the tree could have separated this original combination of markers.

### The Distribution of the Various Phenotypes of NK Cells Defined Groups of HIV-Primary Infected Patients with Different Maturation Profiles

**Figure 2:**
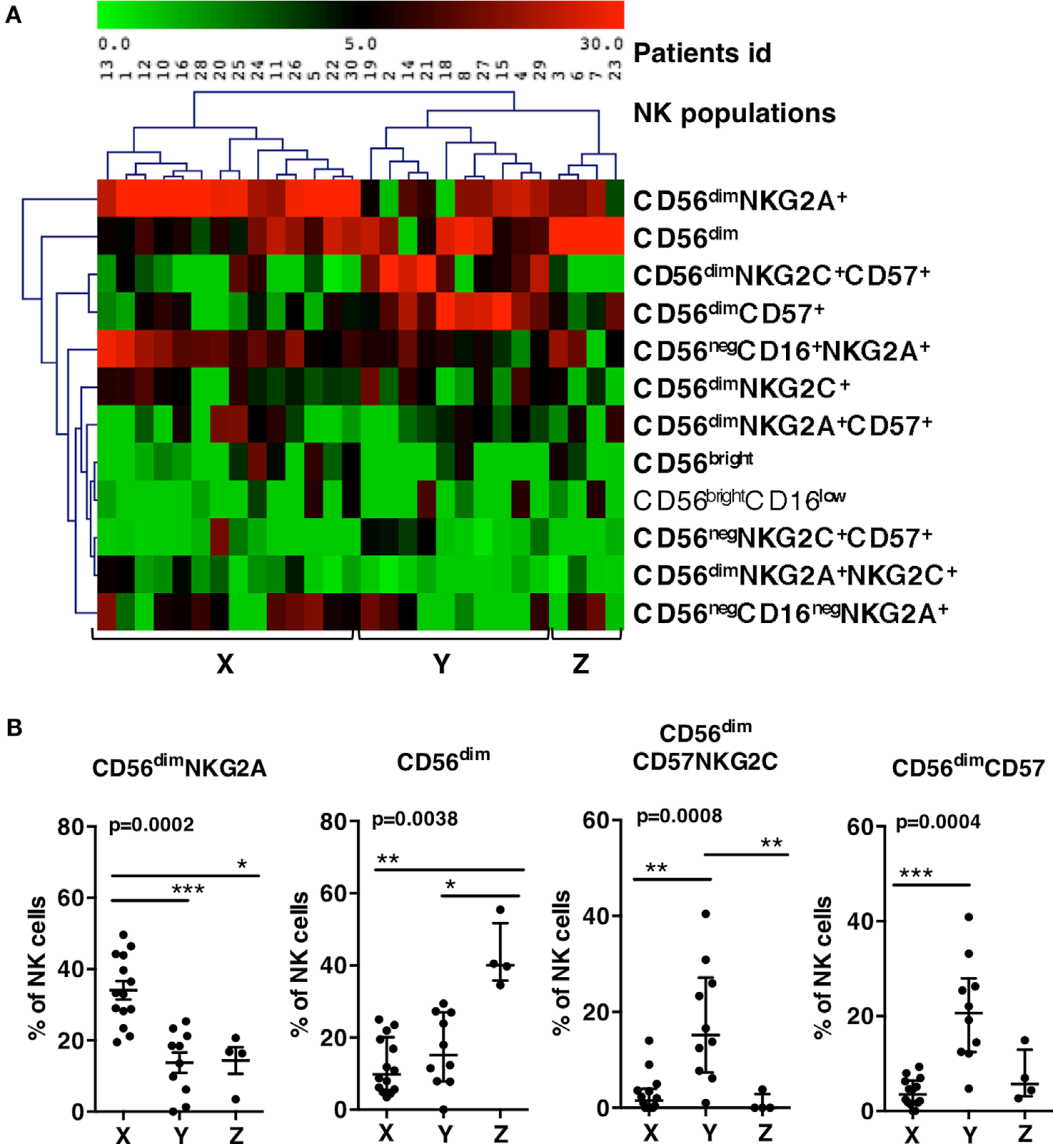
**Patients grouping according to the frequencies of groups of natural killer (NK) populations**. **(A)** Double clustering of the 12 most frequent NK populations shown on right (lines) according to their percentages among NK cells of patients (columns). The squares display the respective frequencies according to the scale of color shown on top. **(B)** Patients X, Y, and Z display significant differences of percentages of CD56^dim^ NKG2A^+^, CD56^dim^, CD56^dim^CD57^+^NKG2C^+^, and CD56^dim^CD57^+^ populations. Errors bars represent the median and the interquartile range. *p*-Values from Kruskall–Wallis test are indicated on top of groups, *p*-Values from Dunn’s multiple comparison posttest on top of pairs: *<0.05; **<0.01; ***<0.001.

We next determined how these NK populations were individually distributed among the 30 primary HIV-infected patients. The blocks of clusters were imported into a spreadsheet program, leading to a matrix with a population name, associated with initial counts of events and patient number. The frequencies of the 12 populations within the total NK cells of each patient were calculated and summarized using pivot tables (Table S1 in Supplementary Material). One population (CD56^neg^CD16^+^CD57^+^), although present in different patients, represented less than 0.5% of total NK cells and was excluded from the analysis. Frequencies of the 12 remaining populations were visualized in a MeV heatmap and used to cluster patients (Figure 2A). Three groups of patients, named X, Y, and Z appeared on the map. Group X was characterized by the highest frequencies of CD56^dim^NKG2A^+^ and CD56^neg^CD16^+^NKG2A^+^ NK cells. Group Y showed high frequencies of CD56^dim^NKGC2^+^CD57^+^and CD56^dim^CD57^+^ NK cells. Group Z contained mainly a population of CD56^dim^ CD57^−^NKG2A^−^ NKG2C^−^ (Figure 2A).

We checked whether the frequencies of the most frequent NK cell populations directing the clustering (the four populations on top of the list) were significantly different between the three groups (Figure 2B). Group X showed significant higher frequencies of CD56^dim^NKG2A^+^ than Y and Z (34% vs., respectively, 13.8 and 14.3%). Group Y showed significant higher frequencies of CD56^dim^CD57^+^ NK cells than X and Z (21% vs., respectively, 2.9 and 7.2%), and higher frequencies of CD56^dim^NKG2C^+^CD57^+^ than X and Z (17.8% vs., respectively, 2.9 and 0.9%). Group Z showed 42.6% of CD56^dim^ NKG2A^−^NKG2C^−^CD57^−^ while this phenotype represented only 12 or 16% of NK cells in, respectively, X and Y. Accordingly, the three groups of patients were significantly characterized by, respectively, high proportion of CD56^dim^NKG2A^+^ for group X, high proportion of CD56^dim^CD57^+^ NK cells for group Y, and high proportion of CD56^dim^ NKG2A^−^NKG2C^−^CD57^−^ for group Z.

### NK Cell Profiles of Patients Correlated with HIV Viral Load at Inclusion

**Figure 3:**
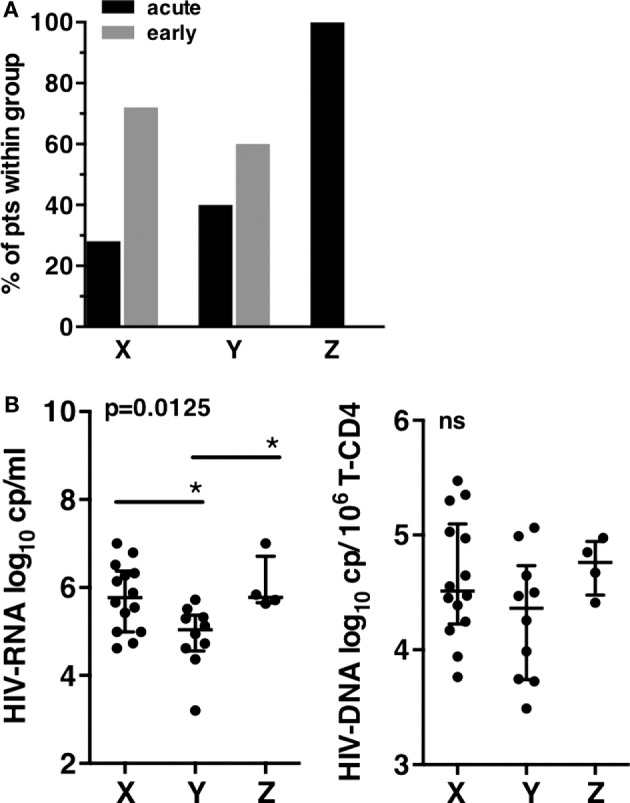
**Clinical and virological characteristics of patients groups at T0**. **(A)** Primary infection status of patients according to the X, Y, and Z groups. **(B)** Human immunodeficiency virus (HIV) viral load. Plasma HIV-RNA (left), HIV-DNA copies associated to millions of T-CD4 cells (right). Error bars represent the median and interquartile range. *p*-Values from Kruskall–Wallis are indicated on top of groups, *p*-Values from Dunn’s multiple comparison posttest on top of pairs: *<0.05; **<0.01; ***<0.001.

To evaluate the relationship between NK profiles of patients and HIV infection, the clinical and virological characteristics of patients within groups were compared at T0 (Table 2). Primary infection was defined by detectable plasma HIV-RNA and incomplete HIV-1 western blot, acute infection by one band or fewer (21) and early infection by more than one band. Four out of 14 patients from group X, 4 out of 10 patients of group Y, and all 4 patients of group Z were acutely infected (Figure 3A). The mean age of patients from each group was not significantly different (37, 40, and 42 years for, respectively, X, Y, and Z), nor was the mean of estimated time since infection and enrollment (36.5, 35.3, and 34.7 days for, respectively, X, Y, and Z) (Table 2). T-CD4 counts were similar for all groups, although group Z patients showed a trend to lower levels (Table 2). T-CD8 counts were significantly higher in X than Y and Z (mean of 2,148 counts/μL vs. 1,308 and 1,415 for, respectively, Y and Z) (Table 2).

**Table 2:** **Groups characteristics**.

	Groups of patients		

Characteristics	X	Y	Z
Number of patients	14	10	4
Acute (% in the group)	28	40	100
Time between estimated date of infection and enrollment (days)	36 (23–55)	35 (22–46)	35 (32–41)
Age (years)	37 (23–64)	40 (23–62)	42 (24–55)
CD4 counts (count/μL)	549 (323–1,012)	584 (368–864)	430 (341–513)
CD8 counts (count/μL)	2,148 (502–8,157)	1,308 (417–2,716)	1,415 (1,140–1,966)
CD4 to CD8 ratio	0.41 (0.08–1.3)	0.57 (0.23–1.1)	0.31 (0.2–0.35)
Human immunodeficiency virus (HIV)-RNA (log_10_ cp/mL)	5.77 (4.6–7)	4.88 (3.2–5.7)	6.05 (5.6–7)
HIV-DNA (log_10_ cp/10^6^ peripheral blood mononuclear cell)	3.8 (3.2–4.7)	3.5 (2.8–4.3)	3.9 (3.2–4.5)
Progenitors [% of natural killer (NK)]	7.7 (3–20)	4 (0–13.9)	6.3 (0.9–9.5)
Effectors (% of NK)	49.2 (29–66)	20.8 (8–28)	0
Intermediate (% of NK)	17.5 (3.5–29)	22.7 (10–39.5)	45.7 (40.5–56)
Mature (% of NK)	6.8 (0–16)	39 (21–54)	8.2 (2.7–15)
Dysfunctional (% of NK)	15 (5.7–41)	10.6 (3.2–21.6)	11.7 (2.3–16.3)

*T0 mean values and (range) are indicated*.

A striking difference was observed when the viral load was compared between the three patient groups. Patients from groups X and Z displayed significantly higher mean HIV-RNA levels than patients from group Y (respectively, 5.77 log_10_ HIV-RNA copies/mL and 6.05 log_10_ HIV-RNA copies/mL vs. 4.88 log_10_ HIV-RNA copies/mL in group Y), while HIV-DNA were not significantly different despite a trend to lower levels for Y (Figure 3B). Thus, the major differences observed between the three groups of patients at inclusion highlighted the lower viral load of group Y, the group defined by an expansion of CD57^+^ NK cells.

### Patients with High Frequencies of Mature NK Cells Displayed Better Immunological Parameters at Inclusion

**Figure 4:**
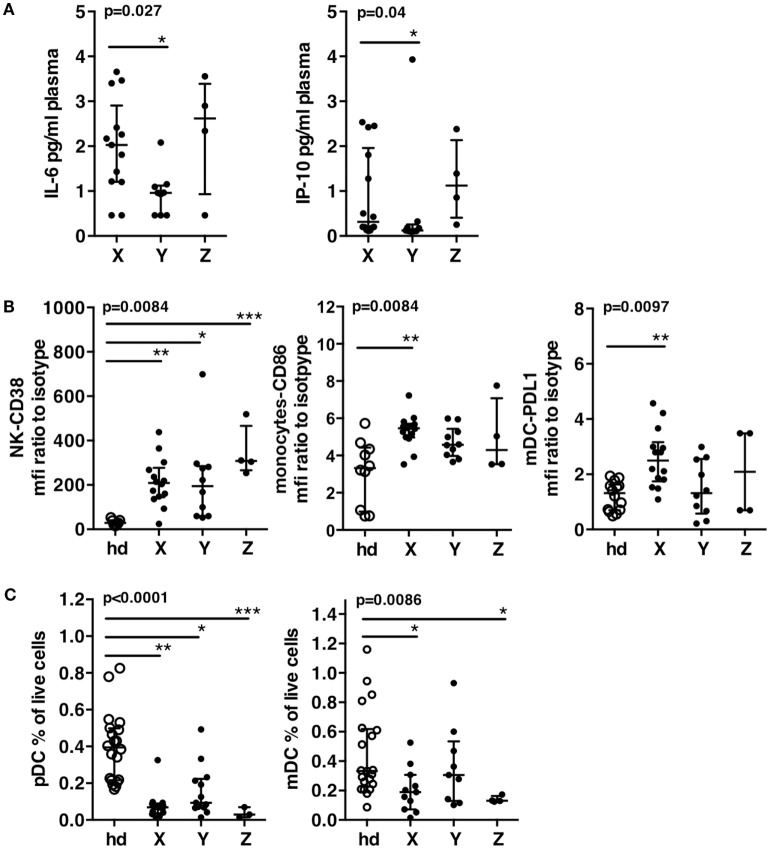
**Immune status of patient groups at T0**. **(A)** Plasma levels of IL-6 and of IP-10. **(B)** Innate immune cells activation of patients groups and a group of 15 healthy donors. CD38 expression on natural killer cells, CD86 expression on monocytes, PDL-1 expression on mDC. **(C)** Dendritic cell exhaustion. Frequencies of pDC’s and mDC’s in the patients groups and in a group of 15 healthy donors. Errors bars represent the median and interquartile range. *p*-Values from Kruskall–Wallis test are indicated on top of groups, *p*-Values from Dunn’s multiple comparison posttest on top of pairs: *<0.05; **<0.01; ***<0.001.

We next addressed the question of whether patients of group X, Y, and Z were different with respect to other major disease progression markers. We analyzed plasma inflammatory markers (IL-6, IP-10) (25, 26) and also focused on markers of innate immune activation and exhaustion, including CD38 expression on NK cells, CD86 expression on monocytes, PDL-1 expression on mDC (27), and pDC and mDC frequencies (28). These markers were all described to be linked to viral load and disease progression (29). They were compared between the groups of patients and some of them were compared to a group of 15 healthy donors (Figure 4).

Patients of group X displayed higher IL-6 and IP-10 plasma levels than patients of group Y (Figure 4A). CD38 expression on NK cells was significantly increased in all patients groups as compared to healthy donors. CD86 on monocytes and PDL-1 on mDC were significantly increased in patients X as compared to healthy donors, while patients Y showed a trend to lower increase and values of PDL-1 not different from that of healthy donors (Figure 4B). All patients groups showed significant decrease of pDC as compared to healthy donors, but patients Y showed a clear trend to higher values of pDC frequencies (Figure 4C). Only patients X and Z showed significant decrease of mDC frequencies while patients Y exhibited values similar to healthy donors (median of, respectively, 0.3 vs. 0.33%) (Figure 4C). Therefore, in addition to exhibit the lowest activation, Y patients displayed low exhaustion of DC, as shown by a trend to higher frequencies of pDC and frequencies of mDC not different from that of healthy donors (Figure 4C). As expected according to their high viremia levels, patients from groups X and Z displayed higher immune activation and exhaustion while patients Y exhibited a better immune status in PHI.

### Patients with High Frequencies of Mature NK Cells Displayed a Lower Viral Load after 3 Months of cART

We then assessed the impact of the different virological and immunological status at inclusion on early cART outcome. While all patients showed a 3-log decrease of viral load after 3 months of cART (Table 2), we searched for those patients having reached a viral load below 50 copies/mL at M3. Notwithstanding the cART regimen, 6 out of 14 (57%) patients X, 1 out of 4 (25%) patients Z, and 7 out of 10 (70%) patients Y reached a viral load below 50 copies/mL at M3. Taken together, only 38% of patients lacking significant frequencies of CD57^+^ NK cells (X + Z) reached a threshold of 50 log_10_ HIV-RNA copies/mL at M3 whereas 70% of patients with high frequencies of CD57^+^ NK cells (Y) could reach it.

### Early cART Modestly Modify the Frequencies of Mature CD57^+^ NK Cells at M3

**Figure 5:**
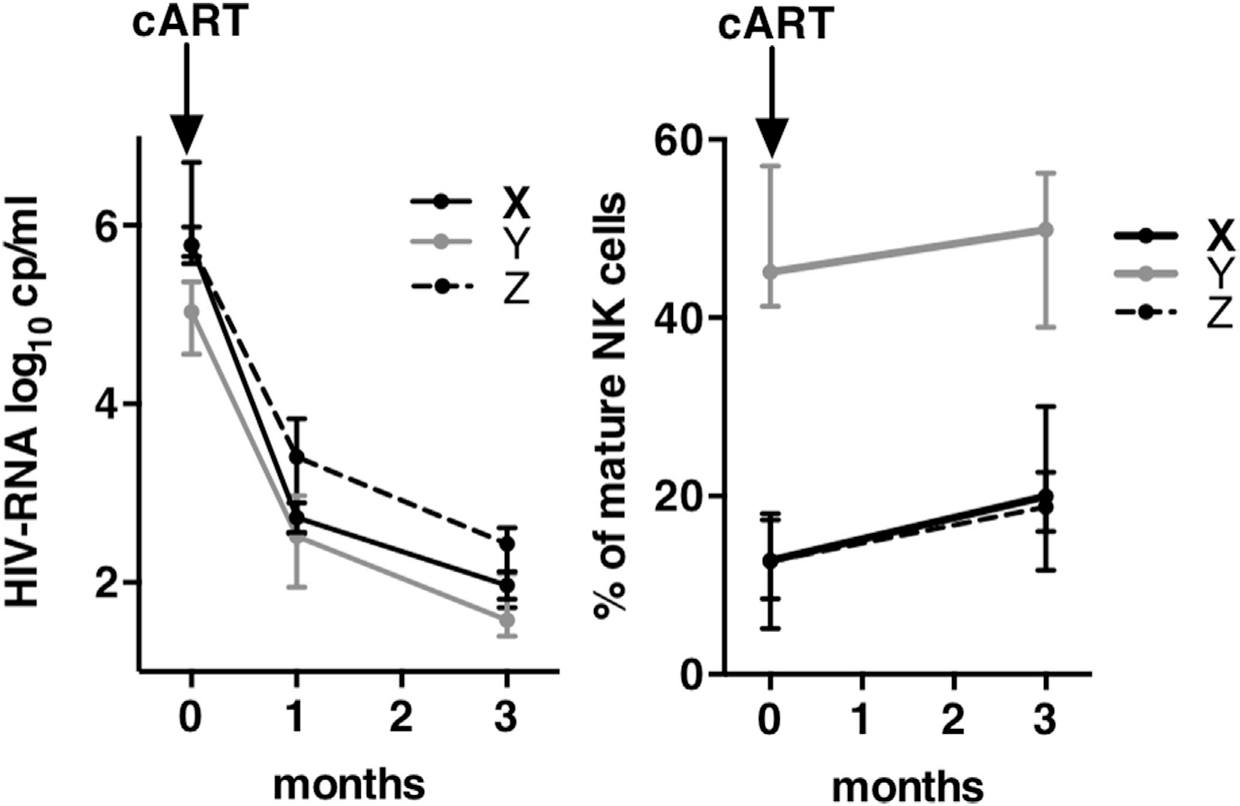
**Kinetics of viral load and of mature natural killer (NK) cells frequencies during 3 months of combination antiretroviral therapy (cART)**. Left graph shows the kinetics of human immunodeficiency virus-RNA between T0 and M3 after cART onset. Right graph shows the kinetics of the frequencies of mature NK cells between T0 and M3. Errors bars represent the median and interquartile range.

In order to evaluate the kinetics of NK cell maturation at a short time after infection, we compared the frequencies of CD57^+^ NK cells at inclusion and after 3 months of cART in groups of patients X, Y, and Z (Figure 5). All CD57^+^ NK cells were summed. HIV-RNA showed a mean decrease of 2 logs in both groups after 1 month of treatment, one additional log being lost during the third month (left graph). In the meantime, the frequency of CD57 NK cells remained unchanged in patients from group Y (47.5% at T0 vs. 48.9% at M3), while the frequencies of CD57 populations of patients from group X and Z significantly increased, or alternatively the proportion of immature NK cells decreased (13.9% at T0 vs. 20.5% at M3 for X; 11.7 vs. 19.4% for Z). Therefore, even if the frequencies of CD57 NK cells increased during the 3 months of cART, the values reached remained far from those of patients from group Y at T0 (47.5%).

## Discussion

Natural killer cells are potent effectors of the innate immune system and key actors in the race engaged between the virus and the host. NK cell compartment is constituted by populations more or less advanced on the maturation pathway, which compose a unique landscape at an individual level. We studied the NK cell compartment of HIV-infected patients at the time of primary infection. In spite of limitations such as the number of patients and the lack of samplings before infection, our results globally support a link between NK cell compartment skewed toward maturation and decreased levels of viral load and immune activation at the time of the primary HIV infection.

While multiparametric cytometry allows deep investigation of human immune cells, discrete subsets resulting from unexpected combinations of markers are found by chance using classical manual analysis. Computation, through the consideration of all parameters at the same time allowed exploration of the full dataset and finding discrete subsets in an unsupervised approach (22). The description of the NK cells of patients into 12 subsets, whereas only six would have been searched according to previous reports (CD56^bright^, four populations of CD56^dim^ and CD56^neg^), provides an added value to the comprehension of the ongoing interplays between NK cell and HIV. One example is the finding of four different CD56^neg^ subsets and their clustering near four different NK cell populations, including CD56^bright^. This result suggests that the defect affecting the NK cell can touch the lineage at different levels of maturation.

Among 12 NK populations, four co-expressed NKG2C in various combinations spanning the whole NK maturation process. The clustering of the CD56^dim^NKG2A^+^NKG2C^+^ subset near CD56^bright^CD16^low^, previously described at the transition between CD56^bright^ and CD56^dim^ (30), suggests they originate from the progenitor. NKG2C^+^ NK cell expansion was reported previously in HIV primary infection (10, 11, 16) and described during Hantavirus and Chikungunya acute infections (12, 31), always in the context of underlying CMV co-infection. The identification of discrete NKG2C^+^ NK cells subsets with a phenotype close to the NK cell progenitor suggests an ongoing generation during primary HIV infection.

The individual distribution of those 12 NK cell populations permits constitution of homogeneous and significantly different groups of patients. Group X included 14 patients whose NK cells were mainly composed of CD56^dim^NKG2A^+^, group Y included 10 patients with high frequencies of CD56^dim^CD57^+^ and CD56^dim^CD57^+^NKG2C^+^, and group Z included four patients whose NK cells displayed a CD56^dim^NKG2A^−^NKG2C^−^CD57^−^ phenotype. To understand the role of those differences on HIV primary infection, clinical, virological, and immunological characteristics of the patients groups were compared. Kinetics of HIV infection could have been involved, but this hypothesis was not sustained by the comparison of the estimated time since infection or by the status of antibodies developed to HIV, defining an acute or early primary infection. The striking difference was the viral load at inclusion: patients with high frequencies of CD56^dim^CD57^+^ NK cells (group Y) had significantly lower levels of HIV-RNA than patients with highest frequencies of CD56^dim^NKG2A^+^ (group X) or CD56^dim^NKG2A^−^NKG2C^−^CD57^−^ NK cells (group Z). Although the low number of patients limits the significance of some comparisons, patients Y, as expected according to their lower levels of HIV-RNA, showed lower immune activation and lower exhaustion of immune cells than patients X and Z. cART initiated in this better environment resulted in increased efficiency at M3. Interestingly, one of the 10 patients showing high frequencies of mature NK cells at inclusion became later a PTC (21).

CD57 is a marker of senescence highly correlated to expression of self-KIR that identifies fully mature NK cells (4). Immature KIR^−^ NK cells mostly express NKG2A to regulate their activity, only a small subset of NK cell co-express NKG2A and KIR. Accordingly, CD57^+^ and NKG2A^+^ NK cells were considered as covering, respectively, mature and immature NK populations. Accordingly, the balance of the NK cell compartment maturation is indeed the difference between the groups: NK cell of patients Y are mainly mature CD57^+^ cells whereas most NK cells of patients X are immature. Thus, the NK cell maturation profile at the time of primary infection appears to be a pertinent marker of a better immune status and response to cART started at the primary infection.

The status of the NK cells before HIV infection is not known. Because NK cell maturation is a dynamic process, imbalance of the NK cell compartment toward maturity or immaturity could be a rapid early consequence of HIV infection. CD57^+^ NK cells proliferate only slightly (4). The dynamics of their generation proposed by the follow-up of NKG2C subset during CMV reactivation in transplant patients (32) suggested that NKG2C^+^CD57^+^ resulted from the contraction of effector NKG2C^+^ several months after control of CMV viremia. During the 3 months of cART, while HIV viremia was controlled, the frequencies of CD57^+^ NK cells slightly increased in the Optiprim patients with low levels at inclusion but remained far below the values exhibited by patients with high frequencies at inclusion, suggesting that the dynamics of NK cell maturation is slower than that of viral load. An expansion of CD56^dim^ was reported during HIV primary infection (14). According to the inverse relationship between proliferation and maturation, this expansion should result in decreasing even more the frequencies of mature CD56^dim^CD57^+^. Taking into account the limited proliferation of mature CD56^dim^CD57^+^ and the slow dynamics of their generation, it is reasonable to speculate that the imbalance of the NK cell compartment toward maturation found at inclusion corresponded to its status at the time of HIV infection, whereas the imbalance toward immature NK cells might be a consequence of early NK cell expansion.

Accordingly, mature CD57^+^ NK cell already present at the time of HIV infection must contribute better to immune control of the virus than CD56^dim^NKG2A^+^ NK cells does. As demonstrated in the NKG2C/CMV model, memory-like NKG2C^+^CD57^+^ NK cells expanded after CMV reactivation are potent producers of IFN-γ (8). Accumulation of NK cells expressing self-KIR was demonstrated in HIV PTCs of the Visconti study (33). Those NK cells were potent producers of IFN-γ upon stimulation with HIV-infected targets, suggesting that this function was crucial for virus control. IFN-γ can induce maturation and activation of T cells, DC, and macrophages (3), which cooperate in virus control. Indeed, DC frequencies in patients with high frequencies of mature CD57^+^ NK cells were similar to healthy donors, suggesting indeed that innate immunity overall was involved.

Paradoxically, a prospective study on a cohort of prostitutes demonstrated an increased risk of HIV infection among those displaying highly diverse NK cell repertoire before infection, diversity being intrinsically linked to maturation (20). The high efficiency of NKG2A^+^ NK cell to kill HIV-infected CD4-T cell targets *in vitro*, recently demonstrated (34) is an underlying mechanism that could be involved in the decreased susceptibility to HIV infection of prostitutes displaying an immature NK cell compartment before infection. Taken together, these observations suggest that if immature NK cells are efficient at the time of infection, once infection is established, other mechanisms involving mature CD57^+^ NK cells contribute better to virus control.

Natural killer cell diversity increases with aging and the number of stimulations encountered, resulting in accumulation of mature NK cells heterogeneous with respects to functional activating and inhibitory receptors (20). Groups of patients showed similar mean age, their NK cell profiles should rather be attributed to individual histories resulting in a unique shaping of their NK cell repertoire. NKG2C^+^CD57^+^ NK cell could be considered as a particular subset of the mature CD57^+^ NK cell compartment whose ligand is known. Increased expression of HLA-E on HIV-infected cells suggest a possible mechanism involving NKG2C+ NK cells (35). Other subsets of the mature CD57^+^ pool might share unknown receptor specificities diversely able to target HIV-infected cells. Besides their functional receptors characteristics, NKG2C^+^CD57^+^ NK cells expanded after CMV infection are now considered as a memory-like or adaptive form of NK cells (8, 13). Recently, this subset has been characterized by a low expression of FcRγ (36) and enhanced potential for broad antiviral responses in the presence of virus-specific antibodies (37). While we cannot identify unambiguously adaptive NK cells, the CD57^+^ NK cells found in patients partially controlling their viral load certainly overlap them. Irrespective of the various mechanisms possibly underlying the contribution of mature NK cell to HIV control, once infection is established, evaluation of this parameter during the primary HIV infection appears relevant in the search for prognostic markers to monitor HIV-infected patients.

## Author Contributions

AC, LM, and CR were the chief investigators of the OPTIPRIM study. AC, CG, and GP enrolled the patients. LM coordinated the data collection. DO and FG-R conceived and designed the innate immunity sub-study. FG-R and FM performed the multiparametric cytometry experiments. GB and SG developed bioinformatics tools of analysis. VA-F and CR were responsible for the virological investigations. CL, MM-T, and MP were responsible for immune investigations. FG-R, DO, AC, CR, and AM interpreted the data. FG-R generated the figures and tables. FG-R and DO wrote the paper. All authors reviewed, revised, and approved the final manuscript. AC, CR, LM, VA-F, and DO were part of the OPTIPRIM scientific committee.

## Conflict of Interest Statement

The authors declare that the research was conducted in the absence of any commercial or financial relationships that could be construed as a potential conflict of interest.

## Abbreviations

PBMCs, peripheral blood mononuclear cells; NK cell, natural killer cell; PTC, posttreatment controller; KIR, killer-cell immunoglobulin-like receptor; HIV, human immunodeficiency virus; PMT, photo-multiplicators.
