# Supplementary material for: A Mature NK Profile at the Time of HIV Primary Infection Is Associated with an Early Response to cART
Source: Front Immunol. 2017 Feb 10;8:54. doi: 10.3389/fimmu.2017.00054 (PMC5300971; doi:10.3389/fimmu.2017.00054)
Supplement: Supplementary file 1 [file Table_1.PDF]

pts ID	Nkbright	NKdim2c+57+	NKdim57+	Bright16+	NKdim2a+57+	CD56neg16+	NKdim2c+	CD56-16-2a+	NKdim2a+	CD56neg2c +57+	NKdim2c+2a+	Nkdim
**1**	0	4.97	1.7	0	9.59	0	26.64	2.86	39.69	1.04	5.78	4.89
**2**	0	40.4	12.49	0	3.08	0	8.05	11.65	1.27	4.59	0.41	17.99
**3**	6.9	3.8	6.98	2.3	6.04	2.29	19.91	0	16.85	0	0.35	34.54
**4**	0	12.4	19.1	9.49	11.67	5.10	2.31	0	25.36	1.26	1.64	11.47
**5**	9.86	3.6	1.4	8.67	3.41	0	6.4	17.68	32.93	0	0	10.89
**6**	3.78	0	2.68	0	1.30	7.11	14.88	9.18	16.39	0	2.95	39.75
**7**	0	0	4.35	8.25	4.12	0	0	15.29	20.68	0	0	40.50
**8**	7.12	1.02	25.4	2.61	1,00	6.86	4.6	2.39	18.48	0	0	29.46
**9**	ND	ND	ND	ND	1.9	ND	ND	ND	ND	ND	ND	ND
**10**	2.46	2.3	9.3	0	5.87	0	18.57	7.51	44.24	0	2.24	5.62
**11**	4.68	1	1.8	0	3.68	8.96	9.80	12.45	19.53	0	2.42	23.52
**12**	1.57	3.4	6.25	0	12.78	3.17	20.95	0	33.32	0.89	1.79	11.82
**13**	0	2	2.41	1.72	8.87	0	28.7	17.62	23.45	0	6	6.17
**14**	0	25.9	22.09	0	9.95	2.87	14.93	5.69	13.59	3.75	0	0
**15**	0	7.74	33.15	0	2.12	3.25	3.98	0	23.34	0	0	7.89
**16**	1.93	0	6.97	0	4.93	7.46	13.64	5.91	46.40	0	4.76	7.96
**17**	ND	ND	ND	ND	ND	ND	ND	ND	ND	ND	ND	ND
**18**	3.45	13.7	40.87	0	0	4.36	8.14	0	0	1.17	0	26.94
**19**	0	16.5	4.73	0	15.61	0	7.74	15.7	6.23	4.77	0	23.87
**20**	0	0	0	0	0	16.8	14.63	5.33	28.24	16.83	3.13	8.78
**21**	0	30.8	12.12	12.32	5.46	3.6	7.48	0	9.81	4.98	1.55	7.74
**22**	3.1	0.41	7.96	0	3.89	0	4.97	7.35	43.92	0	0.64	25
**23**	0	0	14.91	0	1.07	10.29	5.23	1.26	3.48	1.50	0.91	55.43
**24**	15	9	4.58	3.6	4.36	5.69	14.8	0	21.15	1.60	2.09	16.89
**25**	4.1	14	2.1	0	9.24	17.24	10.05	0	28.79	2.593	0.85	4.48
**26**	0	0	5.1	0	4.45	3.65	18.58	15.16	29.04	0	4.48	19.49
**27**	0	6.17	26.25	0	7.10	5.44	7.40	0	18.43	0.52	0.91	27.29
**28**	0	0	0	1.7	0	0	14.28	10.37	49.65	1.42	2.08	3.50
**29**	0	23.3	14.4	0	5.11	3.65	5.72	9.84	21.26	2.69	0	12.39
**30**	5.4	0	4.6	5.75	3.79	1.96	10.36	6.25	36.52	0	1.74	21.93

Table S1: Percentages of NK cells populations at T0
