# Supplementary figures and images for: A Mature NK Profile at the Time of HIV Primary Infection Is Associated with an Early Response to cART
Source: Front Immunol. 2017 Feb 10;8:54. doi: 10.3389/fimmu.2017.00054 (PMC5300971; doi:10.3389/fimmu.2017.00054)

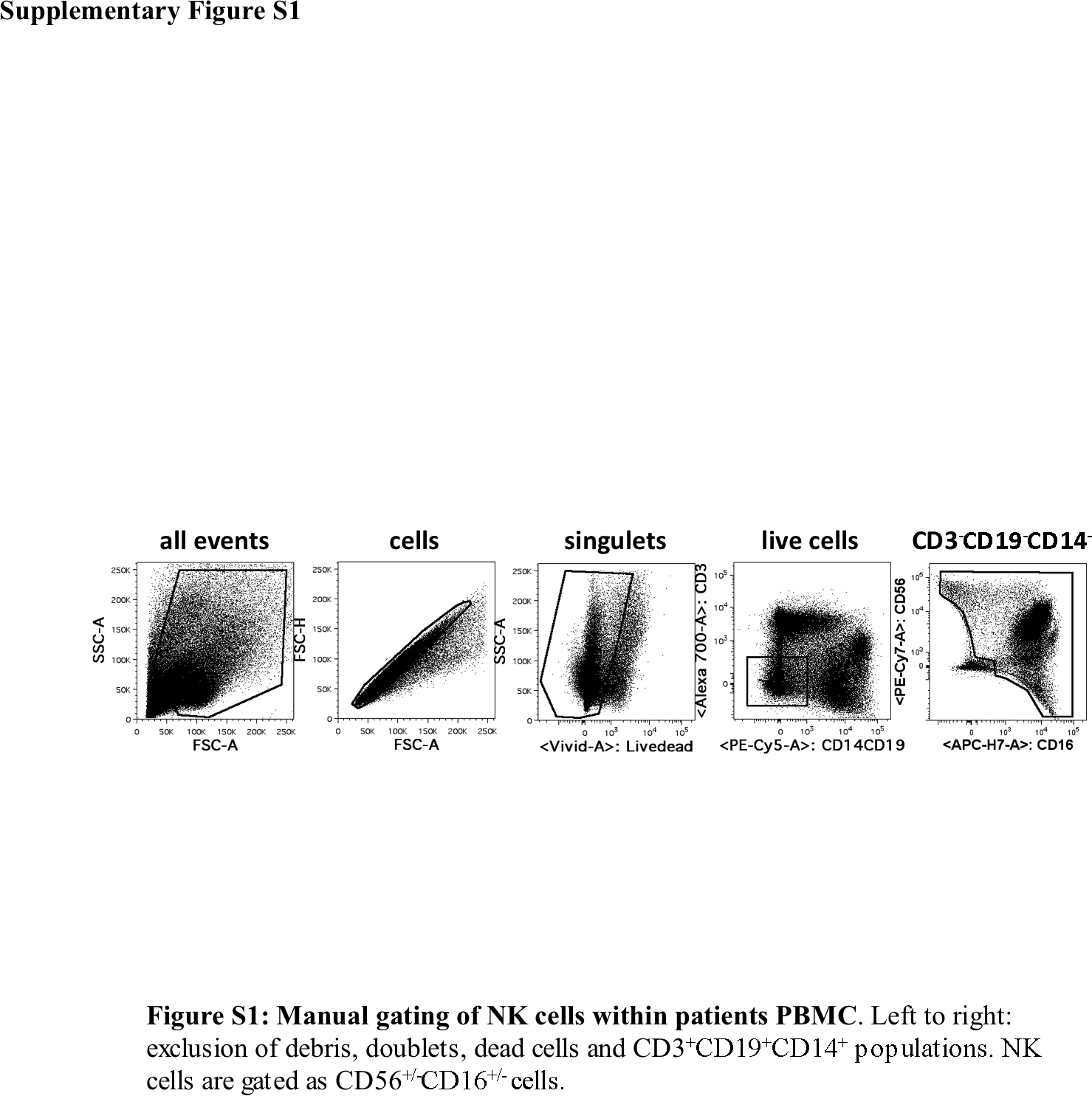

Supplement: Supplementary file 2 [file Image_1.TIFF]
